# Norovirus-induced Gastroparesis

**DOI:** 10.7759/cureus.6283

**Published:** 2019-12-03

**Authors:** Kaylyn N Sawin-Johnson, Clifford D Packer

**Affiliations:** 1 Internal Medicine, Case Western Reserve University School of Medicine, Cleveland, USA

**Keywords:** postviral gastroparesis, gastroparesis, postviral, norovirus, viral gastroenteritis, nausea, early satiety, weight loss, viral

## Abstract

Postviral gastroparesis can result from a variety of viral infections and may cause severe, persistent gastrointestinal symptoms. We report the case of an 85-year-old man with one year of persistent nausea, epigastric pain, early satiety, and 25-pound weight loss after an episode of viral gastroenteritis contracted on a cruise ship. The patient reported that he had tested positive for norovirus shortly after the onset of symptoms. Esophagogastroduodenoscopy revealed no abnormalities, and his symptoms persisted despite treatment for a positive serum H. pylori IgG antibody. Lab workup, including hemoglobin A1c, was otherwise normal, and computed tomography (CT) angiography was unremarkable. A gastric emptying study performed one year after the onset of illness revealed moderate gastroparesis. While most cases of postviral gastroparesis resolve within a year or less, there are a few reports of gastroparetic symptoms lasting two to three years or longer. The pathophysiology might involve a slowly reversible injury to gut neuromodulator cells. Antiviral treatment has not been shown to be effective; symptomatic treatment with antiemetic and prokinetic drugs may be helpful in some cases.

## Introduction

Gastroparesis involves delayed gastric emptying in the absence of mechanical obstruction. Classic presenting symptoms include nausea, vomiting, bloating, early satiety, and abdominal pain. The commonest causes of gastroparesis are diabetes mellitus, postsurgical, and idiopathic, which account for almost 80% of all cases [1]. Less common etiologies include an intestinal pseudo-obstruction, connective tissue disease, Parkinson’s disease, and a variety of viral infections. We report a case of suspected postviral gastroparesis caused by a norovirus infection contracted on a vacation cruise.

## Case presentation

An 85-year-old man with a past medical history significant for gastroesophageal reflux disease presented to the emergency department with a chief complaint of chronic nausea and abdominal pain. The episodes of nausea and associated early satiety had progressed to the point that the patient had lost an estimated 25 lbs. over the preceding four months. The abdominal pain was located in the epigastric region, just inferior to the xiphoid process, and increased to 10/10 in severity with food intake. Esophagogastroduodenoscopy performed six weeks prior to admission showed no significant abnormalities; no biopsies were performed because the procedure was poorly tolerated. He had tested positive for Helicobacter (H.) pylori serum immunoglobulin G (IgG) four months prior and completed a course of lansoprazole, amoxicillin, and clarithromycin. Colonoscopy two years prior revealed no abnormalities. He reported no history of diabetes mellitus or Parkinson’s disease and no prior abdominal surgery, bowel obstruction, or ischemia. Physical examination was significant for a normal abdominal exam and no focal neurologic abnormalities, and laboratory testing revealed a normal hemoglobin A1c. Computed tomography (CT) angiography shortly after admission demonstrated no findings of mesenteric ischemia. His symptoms did not improve with ondansetron 4mg po TID. A gastric emptying study revealed moderate gastroparesis, with gastric retention at one, two, and four hours calculated at 95%, 74%, and 30.5%, respectively (upper limits of normal are 90%, 60%, and 10%).

Upon further questioning, the patient stated that his nausea and abdominal pain had begun about a year before admission when he had gone on a cruise where he had eaten an excessive amount of shrimp, followed by acute onset of nausea, vomiting, diarrhea, and severe epigastric pain. When the symptoms persisted after his return from the cruise, he was evaluated at an outside hospital, where norovirus infection was identified. The patient was unable to recall where he had received care, and thus we were unable to confirm stool reverse transcription-polymerase chain reaction (RT-PCR) or serum antibody testing results. The patient cited this episode on the cruise as the onset of his nausea and abdominal pain and remarked that he had “not felt the same” since.

## Discussion

Although the pathophysiology of postviral gastroparesis is not completely understood, it is thought that the mechanism involves either direct viral damage to the autonomic ganglia or indirect neuronal injury from the inflammatory or immunologic response to the infection [1-3]. Some researchers have localized viral-mediated damage to the interstitial cells of Cajal, either via direct viral injury or an abnormal T-cell immune response. The interstitial cells of Cajal are also referred to as the “pacemaker cells” of the gut. They function to generate spontaneously active currents that drive the mechanical and electrical activities of smooth muscle cells, thus driving the spontaneous rhythmic motility of the gastrointestinal (GI) tract [4-6]. Damage to these cells by inflammatory infiltration or direct viral injury could disrupt normal peristaltic action and result in persistent gastroparesis.

In part, this patient’s presentation closely aligns with other documented cases of postviral gastroparesis: rapid onset of gastroparetic symptoms (typically nausea, vomiting, early satiety, abdominal pain, or weight loss) after an acute viral prodromal phase [4,7-9]. Although norovirus infection is commonly proposed in the literature as a cause of gastroparesis, our MedLine search revealed no other cases of documented norovirus-induced gastroparesis. More commonly implicated agents include parvovirus, cytomegalovirus, Epstein-Barr virus, varicella virus, and herpes family viruses. Unlike this case, the majority of other cases of postviral gastroparesis have been documented in young to middle-aged females [9].

While most cases of postviral gastroparesis have been found to resolve within a year or less (Table 1), there are a few reports of symptoms lasting two to three years or longer [4,9-12]. Many of the documented cases involve a period of sustained symptoms, followed by gradual improvement or resolution. This could indicate a slowly reversible injury to gut neuromodulator cells. The mechanism behind the variable duration of symptoms is unclear. The information gathered from documented cases suggests that age does not correlate with the duration of symptomology. Other possible factors could include differences in the virulence of the various types of viral infection, variations in baseline immune function, or unspecified comorbid conditions. Our patient’s lack of improvement after 12 months, unfortunately, suggests a more chronic illness.

**Table 1:** Comparison to other presentations of postviral gastroparesis * n/v/d: nausea, vomiting, diarrhea

Cases	Age (yrs)	Sex	Acute illness	Duration of Symptoms
Sawin-Johnson & Packer	85	M	Norovirus, n/v/d*, abdominal pain	12 months (active)
Kebede et al. [13]	76	M	Herpes zoster virus	2 weeks
Barkin et al. [14]	66	F	Enterovirus	Unknown
Jaehoon & Chung [11]	59	M	‘Presumed viral’	1.5 weeks
Barkin et al. [14]	57	F	Enterovirus	Unknown
Buppajarntham [15]	52	M	Varicella zoster virus	3 weeks
Naftali et al. [4]	47	F	‘Flu like’	18 months (active)
Barkin et al. [14]	47	F	Enterovirus	Unknown
Thongpooswan et al. [16]	46	F	Cytomegalovirus	3 months
Barkin et al. [14]	44	F	Enterovirus	Unknown
Barkin et al. [14]	42	F	Enterovirus	Unknown
Barkin et al. [14]	39	M	Enterovirus	Unknown
Barkin et al. [14]	36	F	Enterovirus	Unknown
Jaehoon & Chung [11]	36	F	‘Presumed viral’	37 months (active)
Barkin et al. [14]	35	M	Enterovirus	Unknown
Lobrano et al. [17]	31	F	‘Guillain-Barre-like’	1 month (active)
Vassallo et al. [18]	23	F	Epstein-Barr virus, infectious mononucleosis	3 years (active)
Jaehoon & Chung [11]	23	F	‘Presumed viral’	29 months (active)
Jaehoon & Chung [11]	22	M	‘Presumed viral’	4 months
Barkin et al. [14]	21	F	Enterovirus	Unknown
Naftali et al. [4]	19	F	Epstein-Barr virus, sore throat	12 months
Jaehoon & Chung [11]	16	F	‘Presumed viral’	12 months
Jaehoon & Chung [11]	16	M	‘Presumed viral’	12 months
Jaehoon & Chung [11]	16	F	‘Presumed viral’	3 weeks
Yeh et al. [19]	16	F	‘Flu like’	9 months (active)
Yeh et al. [19]	15	F	‘Flu like’	4 months (active)
Naftali et al. [4]	15	F	‘Flu like’	10 months (active)
Naftali et al. [4]	14	M	Vomiting and diarrhea	13 months
Naftali et al. [4]	14	M	‘Flu like’	12 months
Yeh et al. [19]	13	F	‘Flu like’	3 months (active)

As the diagnosis of postviral gastroparesis often relies heavily on history, other causes, as well as the possibility of an idiopathic nature, should be considered. Treatment of postviral gastroparesis can be difficult. Antiviral medications have not been shown to be effective, and specific treatment guidelines are lacking. The main targets of treatment are the alleviation of symptoms with antiemetics and prokinetic agents such as metoclopramide and erythromycin, correction of malnutrition, and resumption of adequate oral intake [9]. There is one report of a 34-year-old woman with severe postviral gastroparesis whose symptoms improved rapidly with mirtazapine treatment [12]. Additional research is needed on the natural history, prognosis, pathophysiology, and optimal treatment of postviral gastroparesis.

## Conclusions

Postviral gastroparesis should be suspected in patients with persistent gastrointestinal symptoms after viral gastroenteritis or other viral syndromes. A subset of patients has gastroparetic symptoms lasting more than one year. Antiviral treatment has not been shown to be effective, but symptomatic treatment with prokinetic and antiemetic agents may be helpful.
